# Multiscale Mechanical Characterization of Polyether-2-ketone (PEKK) for Biomedical Application

**DOI:** 10.3390/bioengineering11030244

**Published:** 2024-02-29

**Authors:** Gianpaolo Serino, Fabio Distefano, Elisabetta M. Zanetti, Giulia Pascoletti, Gabriella Epasto

**Affiliations:** 1 Department of Mechanical and Aerospace Engineering (DIMEAS), Politecnico di Torino, 10129 Turin, Italy; 2 PolitoBIOMed Laboratory, Politecnico di Torino, 10129 Torino, Italy; 3 Department of Engineering, University of Messina, Contrada Di Dio, 98166 Messina, Italy; gabriella.epasto@unime.it; 4 Department of Engineering, University of Perugia, 06125 Perugia, Italy; elisabetta.zanetti@unipg.it (E.M.Z.); giulia.pascoletti@unipg.it (G.P.)

**Keywords:** multiscale mechanics, mechanical properties, nanoindentation, micromechanical characterization, digital image correlation, PEKK, viscous behavior, anisotropy, lattice structure

## Abstract

Polyether-ether-2-ketone (PEKK) is a high-performance thermoplastic polymer used in various fields, from aerospace to medical applications, due to its exceptional mechanical and thermal properties. Nonetheless, the mechanical behavior of 3D-printed PEKK still deserves to be more thoroughly investigated, especially in view of its production by 3D printing, where mechanical properties measured at different scales are likely to be correlated to one another and to all play a major role in determining biomechanical properties, which include mechanical strength on one side and osteointegration ability on the other side. This work explores the mechanical behavior of 3D-printed PEKK through a multiscale approach, having performed both nanoindentation tests and standard tensile and compression tests, where a detailed view of strain distribution was achieved through Digital Image Correlation (DIC) techniques. Furthermore, for specimens tested up to failure, their fractured surfaces were analyzed through Scanning Electron Microscopy (SEM) to clearly outline fracture modes. Additionally, the internal structure of 3D-printed PEKK was explored through Computed Tomography (CT) imaging, providing a three-dimensional view of the internal structure and the presence of voids and other imperfections. Finally, surface morphology was analyzed through confocal microscopy. The multiscale approach adopted in the present work offers information about the global and local behavior of the PEKK, also assessing its material properties down to the nanoscale. Due to its novelty as a polymeric material, no previous studies have approached a multiscale analysis of 3D-printed PEKK. The findings of this study contribute to a comprehensive understanding of 3D-printed PEKK along with criteria for process optimization in order to customize its properties to meet specific application requirements. This research not only advances the knowledge of PEKK as a 3D-printing material but also provides insights into the multifaceted nature of multiscale material characterization.

## 1. Introduction

The PAEK (polyaryletherketone) family of thermoplastic materials is characterized by its excellent mechanical properties [1,2], and it has found various applications, especially in the spatial and biomedical fields. PEEK and PEKK [3,4] are two members of this family; they both have aromatic rings but differ in the ratio of ether and keto groups. PEKK usage is more recent, and the interest towards this material stems from its exceptional mechanical strength, biocompatibility, bioactivity, and resistance to wear and corrosion [4,5,6]. The elastic modulus of PEKK is much lower than the elastic modulus of titanium, being very close to dentine, cartilage, and bone. These properties make it an ideal choice for various biomedical applications [7], including orthopedic applications [8], intervertebral devices [9], mandibular prostheses [10], hip prostheses [11], and dental implants [12,13,14], where the use of PEKK gained significant attention in recent years, showing promising clinical outcomes [15,16].

The production of PEKK devices through additive manufacturing (AM) has brought further advantages, since this process enables the creation of intricate designs and customizable implants with high accuracy, tailored to specific patient needs [17,18]. In addition, AM allows for the fabrication of porous structures within PEKK implants, which promote osseointegration [19,20].

The employment of additively manufactured PEKK prostheses poses some major challenges, since these devices are qualified as Class III Medical devices, and therefore require the setting up of a strict certification workflow [21]. One major step in this process is the proper characterization of the mechanical behavior of this material and its surface properties. As is well-known, the success of an endoprosthesis is strictly related to its biomechanical performance [22] and superficial properties [23]. The first one guarantees, on one side, the capability of the prosthesis to withstand physiologic loads and, on the other side, a balanced distribution of loads between the bone and the prosthesis, avoiding stress shielding phenomena [24,25] or subsidence [26]. As is well-known, surface properties are fundamental when dealing with osteointegration, where the rugosity of the surface and pore size are key properties [27]. This requirement has led to the development of a new material, PEKK, which has been reported to be a promising orthopedic material thanks to the variety of chemical surface modification it can undergo [4], finally resulting in improved osteointegration ability compared to the more widely used PEEK [28,29,30].

Similarly to PEEK, characterizing the mechanical behavior of PEKK is quite complex due to the interplay of many factors. Previous tests on PEEK from other authors have clearly outlined the viscous behavior of this material and its strain rate dependency, both for solid [31,32,33,34] and lattice materials [35]. 3D-printing production brings some more variables such as printing parameters and building direction. All this considered, the hypothesis of a perfectly uniform strain distribution in uniaxial dog-bone specimens needs to be thoroughly discussed, referring to refined experimental setups for strain measurement such as Digital Image Correlation (DIC) [36].

The physical properties of manufacts obtained through fused deposition modeling (FDM) parts are commonly evaluated by printing dog-bone shaped specimens [37] and setting different values of print parameters such as orientation, velocity, and temperature, with the aim of investigating their influence on the final manufact. In this work, a different approach was followed, namely having fixed manufacturing variables and focusing on the mechanical and geometrical characteristics of the printed material. In particular, the mechanical behavior was defined to try and go one step further than current knowledge, also assessing the material’s performance down to the nanoscale [38]. The multiscale approach adopted here permitted us to obtain information about the global and local mechanical behavior of 3D-printed PEKK, providing comprehensive data for predicting the material’s performance.

To the best of the authors’ knowledge, there is very limited literature on the mechanical properties of PEKK due to its novelty as a polymeric material for 3D printing [39,40,41]. In addition, no previous studies have dealt with the multiscale analysis of 3D-printed PEKK; even more widely used PEEK has been seldom analyzed, following a multiscale approach on 3D-printed specimens, with an exception made for Pérez-Martín et al. [42] and Lu et al. [43], who have performed nanoindentation analyses to characterize new PEEK composites.

In this work, various techniques were explored to investigate the properties of the material. These techniques encompassed macro-scale tests (tensile and compression tests), geometric analyses at microscale through micro-CT and confocal microscopy, as well as nanoscale tests such as nanoindentation. Additionally, fractured areas were examined using Scanning Electron Microscopy (SEM).

## 2. Materials and Methods

### 2.1. Specimens for Macro Tests

The specimens were manufactured through the FDM technique, extruding PEKK Antero™ 800NA-FDM, supplied by Stratasys (Stratasys, Eden Prairie, MN, USA), which is a thermoplastic filament of a semi-crystalline engineering-grade material [2]. The samples were fabricated using a T20F tip and 0.254 mm layer heights on the Stratasys Fortus F900™ 3D printer at a temperature of 200 °C and raster angle of ±45° [2,44]. 

**Figure 1:**
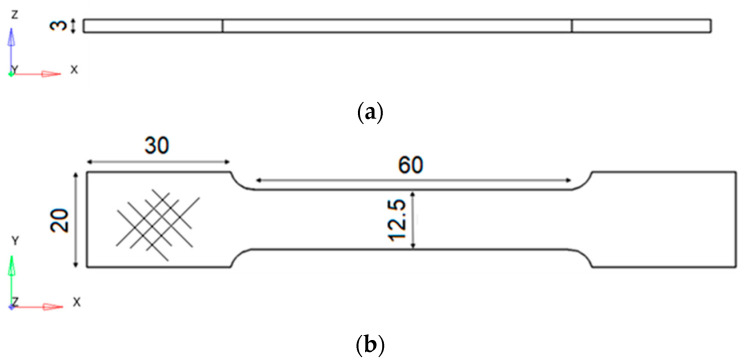
Tensile test specimen. (**a**) View of the xz-plane; (**b**) view of the xy-plane; the raster angle is visible on the left side.

**Figure 2:**
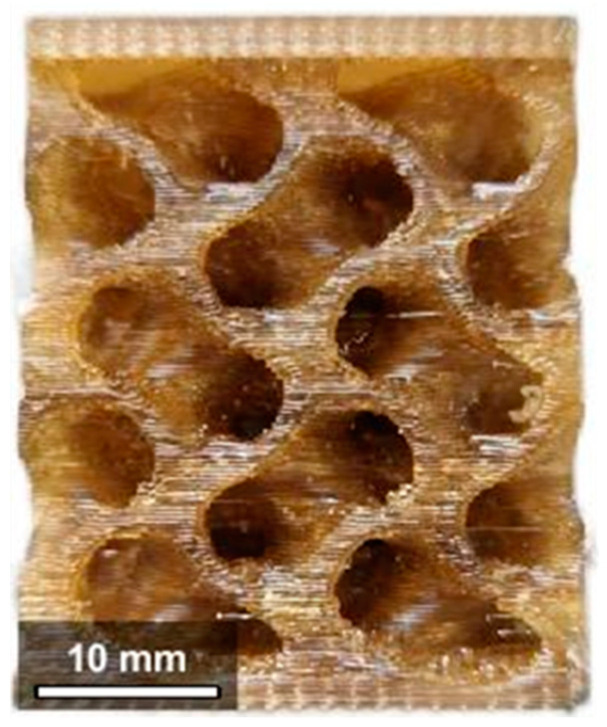
Compression test specimen.

Six dog-bone shaped specimens and four gyroid specimens, respectively, for tensile and compression tests, were manufactured according to the quoted drawings in Figure 1 and Figure 2. Tensile specimens were printed along the *z*-axis (Figure 1a), while each layer on the xy-plane was printed with alternating printing directions at +45° and −45° (Figure 1b).

The specimens for the compression tests were fabricated using a gyroid skeletal lattice with 55% relative density and a unit cell size of 15 mm, according to Li et al. [45]. The specimen’s dimensions are 30 × 30 × 30 mm^3^. Two plates with a thickness of 2 mm were added at the top and bottom faces (Figure 2).

#### Volumetric and Surface Analyses of Specimens

The morphological analysis of gyroid samples was carried out by a micro-computed tomography (μCT), designed and manufactured by the IKTS (Fraunhofer, Dresden, Germany). The X-ray tube was set at a voltage of 160 kV and a current of 0.1 mA. A total of 1600 projections were used in the 3D profile generation. The pitch size was 0.2 μm, with a resolution of 3 μm in the reconstruction step. The acquired CT images were stored in DICOM format for subsequent analysis. μCT analyses were aimed to detect and measure the defects’ volume, as well as to estimate the actual density ratio. The μCT images were manually segmented.

**Figure 3:**
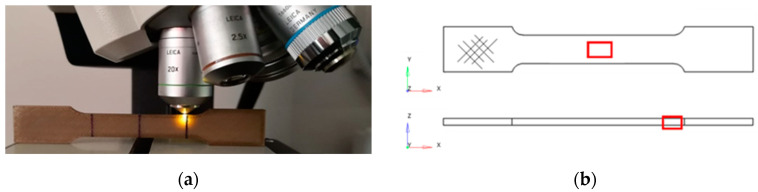
(**a**) Experimental setup to perform confocal microscopy on the lateral surface of the specimen; (**b**) highlighted areas analyzed by the microscope in the xy- and xz-planes.

The surface properties were analyzed through confocal microscopy, performed with a Leica DCM 3D microscope (Leica Microsystems, Wetzlar, Germany), equipped with an EPI 20X-L objective. The experimental setup to perform the confocal microscopy on the lateral side of the specimen (that is, along the *z*-axis, according to Figure 1b) is reported in Figure 3a, while Figure 3b highlights the areas analyzed in the xy- and xz-planes. Both the superior (a 2.06 × 0.87 mm^2^ wide area on the xy plane, Figure 3b) and the lateral (a 1.58 × 1.19 mm^2^ wide area on the xz surface, Figure 3b) surfaces of the tensile specimens were analyzed. On the lateral surface, the major focus was on the transition zone (Figure 3a), where fractures are most likely to occur.

Data reported in Table 1 were obtained by means of LeicaMap 6.2 software added to LeicaScan DCM 3D software, where the *z*-scan covered a height of 180 μm with a *z*-step of 4 μm. The *z*-direction of the confocal microscope represents the vertical displacement of the objective, according to the experimental setup in Figure 3a.

### 2.2. Mechanical Tests at Macroscale

Tensile and compressive tests were carried out by the universal testing machine Italsigma FPF 25, equipped with a 25 kN load cell HBM U10M. Tensile tests were performed according to ASTM D638-14 [46], which defines the standard test method for tensile properties of plastics at six different crosshead speeds, ranging from 0.5 mm/min to 100 mm/min with 250 N preload; referring to the distance between the tensile grips of 76 mm, the displacement rate corresponds to a nominal strain rate ranging from 1.05 × 10^−4^ s^−1^ to 2.18 × 10^−2^ s^−1^. The compressive tests were performed at four crosshead speeds ranging from 0.5 mm/min to 10 mm/min (i.e., a strain rate ranging from 2.18 × 10^−4^ s^−1^ to 4.71 × 10^−3^ s^−1^, referring to the nominal 30 mm height), moving from a 300 N preload up to rupture.

#### Analysis of Strain Distributions

The hypothesis of strain uniformity is questionable due to the non-homogeneity and anisotropy of 3D-printed PEEK. Consequently, the authors made use of Digital Image Correlation to obtain the actual strain distribution through the Open-Source Matlab software Ncorr (version v1.2.2) [47]. No speckle pattern was needed on the specimens, thanks to their respective rugosity and porosity: images were acquired with a resolution of 640 × 480 pixels using a super-macro setting on a Fujifilm HS20EXR digital bridge camera, at a frame rate equal to 30 Hz. An additional neutral led lamp was used to obtain homogeneous lighting on the specimens during the tests.

### 2.3. Micromechanical Characterization

The PEKK sample to be tested through the nanoindentation technique, using the Nanovea CB 5OO nanoindenter (Nanovea, Irvine, Southern California, CA, USA), was extracted from a never-tested dog-bone specimen printed for uniaxial testing. The sample was embedded and finely polished before testing. A 5 × 4 matrix was set to obtain a total of 20 nanoindentation tests. All tests were performed in load control, imposing a loading rate of 15 mN/s, a maximum load of 300 mN, and a holding time of 20 s. The mechanical properties, i.e., the nanoindentation hardness and the nanoindentation modulus, were estimated using the well-know Oliver and Pharr method [48].

### 2.4. Scanning Electron Microscopy (SEM) Analysis

Fracture surfaces of specimens subjected to tensile tests and the shape of the indentation imprints were observed with a scanning electron microscope VEGA (Tescan, Brno, Czech Republic), setting 20 keV as the acceleration voltage and 1 nA as the current, under high vacuum conditions (10^−7^ Pa). The fractured surfaces had to be coated with a 6 nm thick conductive layer of Cr-Pt through a high-resolution sputter, due to the nonconductivity of the PEKK material.

### 2.5. Statistical Analysis of Data

Statistical analysis was performed by applying a non-parametric *t*-test. The significance of all statistical tests has been set to 0.05.

## 3. Results and Discussion

### 3.1. Volumetric and Surface Analysis of Specimens

**Figure 4:**
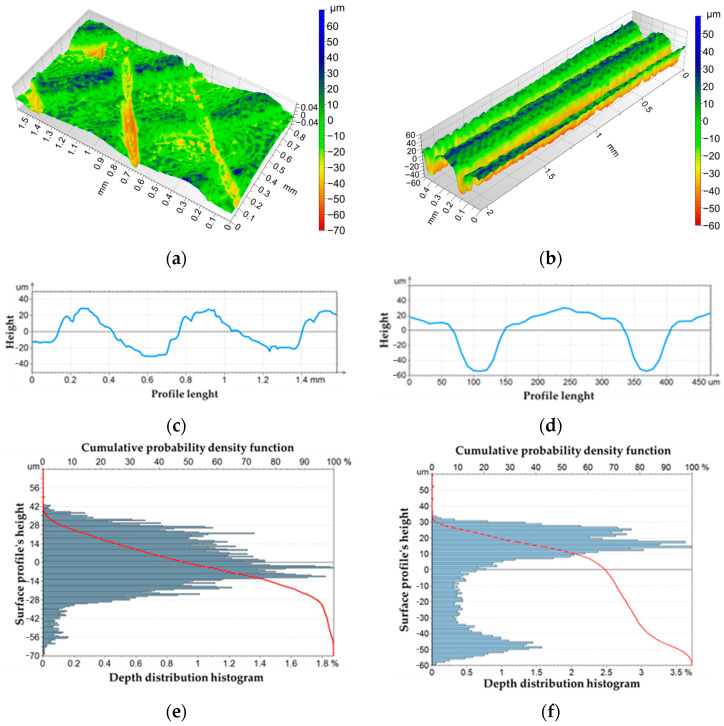
Surface topography of tensile specimen: upper surface (**a**) and lateral surface (**b**). Rugosity profiles of upper surface (**c**) and lateral surface (**d**). Abbott–Firestone curves of upper surface (**e**) and lateral surface (**f**). The red curvers in (**e**,**f**) are the cumulative probability density functions.

The results of the microscopy performed on the upper surface have been reported in Figure 4a,c, while Figure 4b,d,f reports the results of the analysis on the lateral surface. Data reported in Table 1 were obtained according to the ISO 25178 [49] standard. The Ssk parameter, lower than 0, indicates that the height distribution is biased above the mean plane. The calculated values of the Kurtosis parameter (Sku) are a measure of the sharpness of the roughness profile. The upper surface presents a Sku value of 3.04, which indicates that the height distribution is spiked, as evident in Figure 4a. The lateral surface presents a Sku value of 2.23; therefore, the height distribution is smoother compared to the upper surface, as visible in Figure 4b. Considering the calculated values in Table 1 of Sp and Sv, it can be assessed that for the upper surface, the valleys (70.34 mm) are deeper than the peaks (49.23 mm). The lateral surface presents comparable values of peaks and valleys, equal to 66.23 mm and 60.12 mm, respectively. The arithmetical mean height of the surface Sa is an extension of the arithmetic mean height of the profile (Ra). Such a value is used to evaluate the surface roughness, which is 15.12 mm for the upper surface and 22.96 mm for the lateral surface. Previous studies have highlighted that rough surfaces promote cells regeneration in implants made of plastics belonging to the same family of PEKK [50]. Other studies have emphasized how the higher complexity of the PEKK surface may be produced by the higher number of ketone groups and could be beneficial to osteointegration [3].

Regular waves are visible both on the upper and lateral surfaces: the waves on the upper surface are produced by filament deposition at ±45° one next to the other, while waves on the lateral surface correspond to layer deposition. The rugosity profile of the upper surface (xy-plane), reported in Figure 4c, presents waviness with a sinusoidal-like profile and a distance between consecutive peaks or valleys of about 0.6 mm. Figure 4d shows the rugosity profile of the lateral surface (xz-plane), where the distance between two consecutive valleys is 0.25 mm, which is consistent with the layer height equal to 0.254 mm set in the printing process. The Abbott–Firestone curve in Figure 4e shows how the upper surface peaks and valleys concentrated mostly within a range of ±14 µm, while for the lateral surface, they are in the range of 10–30 µm for peaks and reach −50 µm for the valleys (Figure 4f).

The confocal microscopy did not find any singularity that could potentially lead to stress concentration and/or promote crack initiation.

**Figure 5:**
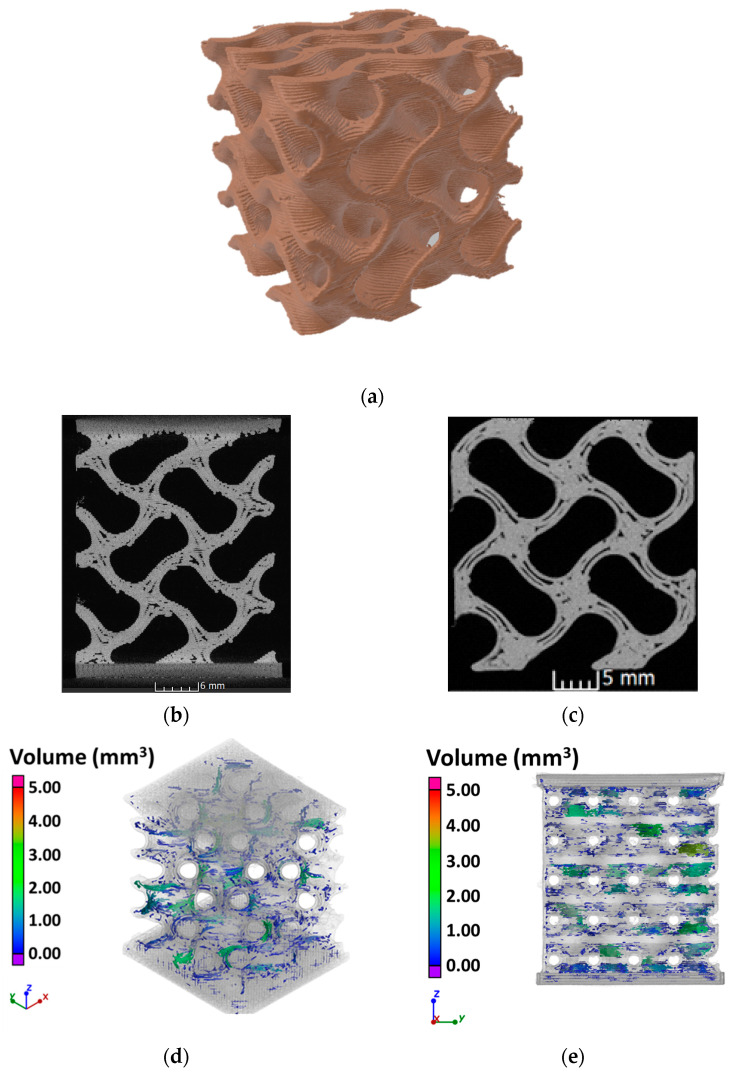
(**a**) Volumetric reconstruction of the specimen; (**b**,**c**) slices showing internal defects; (**d**,**e**) counts of defects’ volume.

The volumetric analysis of the gyroid specimen has produced the results reported in Figure 5, where the distribution and size of defects and the reconstruction of the specimen can be observed. The calculated density ratio was equal to 0.41, with defect porosity reaching 3.5%. The analysis enlightened the structural integrity of the unit-cell walls, which did not experience any discontinuity. Nevertheless, internal defects were detected. Some slices affected by significant voids are shown in Figure 5b,c. Such defects are likely to have been produced by a lack of adhesion between layers during the manufacturing of the specimens due to the twisted geometry of the gyroid cell. The defects were mainly located around the cell holes, and their volumes range between 0–10 mm^3^, with distributions shown in Figure 5d,e.

The volumetric reconstruction also allowed for the assessment of the interconnectivity of the cell’s pores, which is a critical aspect for the osteointegration process as it allows the passage of physiological fluids [51]. The analysis of the reconstructed volume has shown positive results in this sense. This leads to the conclusion that the printed PEKK specimens are indeed good candidates as bone scaffolds [52].

### 3.2. Mechanical Tests at Macroscale

**Figure 6:**
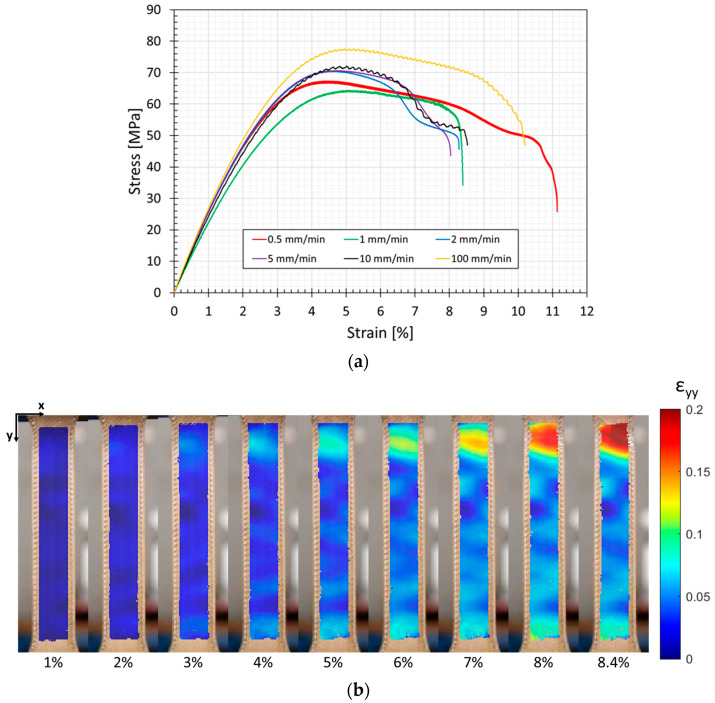
(**a**) Tensile test results: stress–strain curves; (**b**) explanatory strain distribution obtained through DIC analysis, performed for sample tested at 1 mm/min. The color bar reports the strain values along the *y*-axis direction; the percentage values beneath the strain maps indicate the corresponding strain value of the stress–strain curve.

The results of the tensile tests are reported in Figure 6 and Table 2. The elastic modulus was estimated, referring to the initial linear part of the stress/strain curves.

According to Figure 6, once the maximum load was reached, necking deformation takes place, up to the fracture of the specimen.

The curves showed a limited sensitivity of PEKK to the strain rate (for example, if compared to soft tissues’ behavior [53]). The elastic modulus and tensile strength remained very similar in the range of 1 to 5 mm/min, and both increased at a 100 mm/min loading rate, even if variations were below 20%. The statistical analysis of results confirmed that the strain rate was relevant only for the tensile strength (*p* = 0.04) and provided that 100 mm/min data were included.

As reported in Figure 6, for the specimen tested at 1 mm/min, the DIC technique allowed us to calculate the deformation behavior of the specimen subjected to tensile load, in terms of Eulerian–Almansi y-strains [54].

The specimen tested at 1 mm/min is representative of the batch, since all the tested specimens follow the same fracture behavior. In the first part of the stress–strain curves, local strains are almost constant along the gauge length of the specimen (Figure 6 at global strain 1% and 2%). Beyond the elastic limit, local strains start to increase at the top and bottom parts of the specimen, as reported in Figure 6 (global strain equal to 3–4%). After the maximum nominal stress is reached, strains locally increase at the top part of the specimen up to failure, when the global strain reaches 8.4%. Moreover, according to Figure 6, necking takes place at the top region starting from 7% global strain onwards (Figure 6).

**Figure 7:**
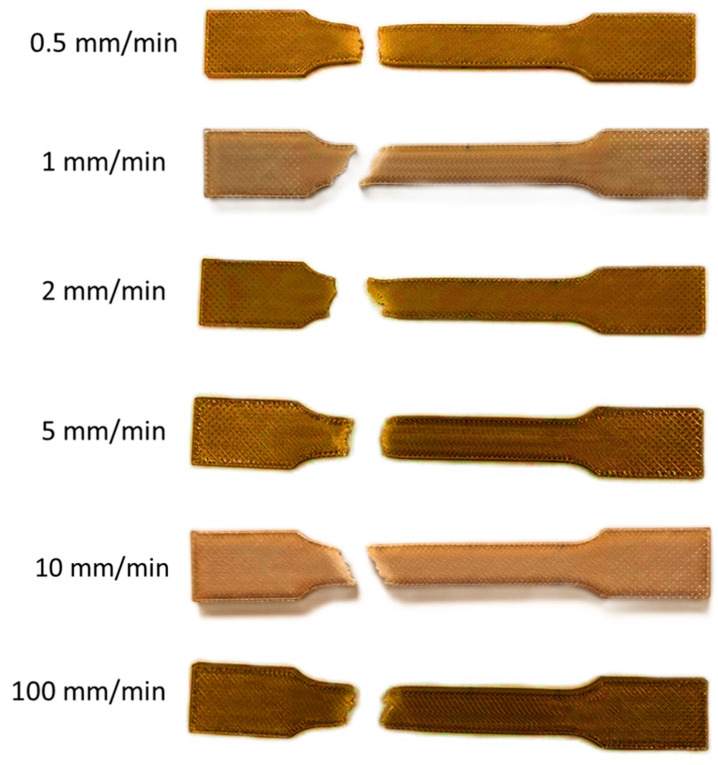
Specimens tested during tensile tests.

Failed specimens are reported in Figure 7. The fracture line generally follows a 45° orientation, which is the orientation of the infill pattern.

From each DIC frame, the mean longitudinal strain value is calculated in the region of interest (that is, the whole frontal area of the gyroid structure), thus obtaining a strain value that was plotted against the respective nominal stress, in order to evaluate the elastic modulus.

The average elastic modulus was found to be equal to 2.55 (±0.15) GPa, which is not significantly different from 2.64 GPa, which is the value reported by Stratasys [44] for specimens printed with Antero 800NA PEKK along the xz direction (layers’ deposition along the specimen width). On the contrary, the elastic modulus of 2.77 GPa, found in the same document, for specimens printed along the zx direction (layers’ deposition along the specimen length) was significantly different (*t*-test *p* < 0.02). The small difference found in the values of the elastic modulus indicates that the printing direction may influence the mechanical properties, in addition to the printing parameters, which are well-known to play a crucial role in determining the final mechanical properties of specimens. Similar tests were performed only in one other study, the results of which reported a significantly higher value for the elastic modulus (5.1 GPa) [55]. This finding leads us to think that the elastic modulus of the 3D-printed manufact is significantly lower when compared to the massive material. Considering the mechanical properties of the massive material, PEKK appears to be closer to bone; nonetheless, it should be considered that bone substitutes should be produced as porous materials to promote osteointegration [52]. Furthermore, other authors analyzing similar specimens, with the same printing orientation but using 3D-printed PEEK found an elastic modulus equal to 1.64 GPa at the same raster angle (±45°) [56]. It is therefore possible to conclude that PEKK is stiffer than PEEK. Thus, PEKK material could be more suitable also from the point of view of the elastic modulus unless a different raster angle is chosen. It should be considered that when using PEEK, moving from a raster angle of ±45° to 0° or 90°, the elastic modulus can vary from 1.64 GPa [56] to 3.79 GPa [57], respectively.

The average tensile strength was found to be equal to 69.0 (±3.09) MPa (not considering the value obtained at 100 mm/min since it was significantly different from the others). This value is not significantly different from the value reported by Stratasys for specimens obtained with the same printing orientation (73.9 MPa), while it is significantly different (*t*-test *p* < 0.002) from the ultimate strength found for specimens printed along the zx direction (59.7 MPa). The ultimate strength reported for 3D-printed material is significantly lower when compared to 115 MPa found by authors who performed tests on massive materials [55]. Both layer thickness and raster angle play a role in determining the tensile strength of 3D-printed poly(aryl-ether-ketone) materials (PAEKs), as demonstrated in a previous work on PEEK [58], which, however, reports lower tensile strength for all parameters combinations. The tensile strength can be improved further by refining the 3D-printing technology. Referring to PEEK, in some studies, very high tensile strength values were reported, such as 89 MPa [56] or 85 MPa [59]. These authors employed very refined 3D-printing technologies: every spool was dried for 8 h before printing at 80 °C, and the chamber, build plate, and nozzle were preheated to 200 °C, 250 °C, and 450 °C, respectively. Surprisingly, those specimens performed very well, exhibiting mechanical properties close to those of PEEK reinforced by carbon fibers [57,59].

Finally, the average elongation at break obtained for the specimens analyzed in this work was equal to 9.1%, which is significantly different from the Stratasys data [44] both for specimens printed along the xz orientation (6.1%; *t*-test *p* < 0.003) and zx orientation (2.3%; *t*-test *p* < 5 × 10^−5^). This result could be ascribed to the printing orientation, speculating that this setting could influence the elongation at break.

**Figure 8:**
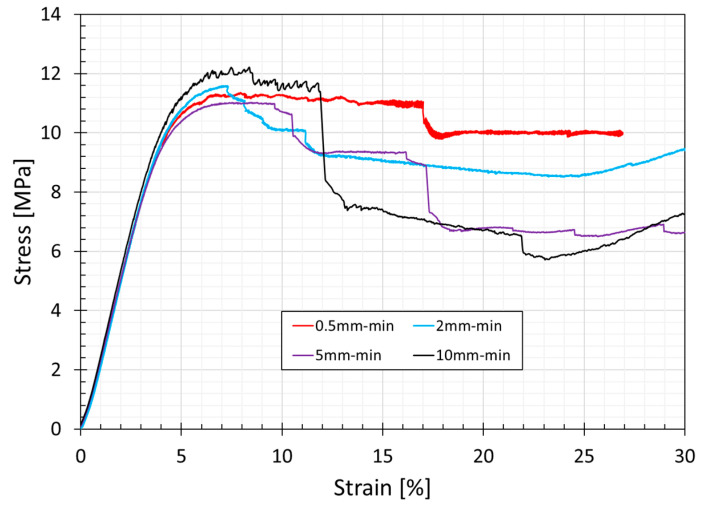
Stress–strain curves obtained from compressive tests performed on cubic gyroid specimens (30 × 30 × 30 mm^3^) at different test speeds.

**Figure 9:**
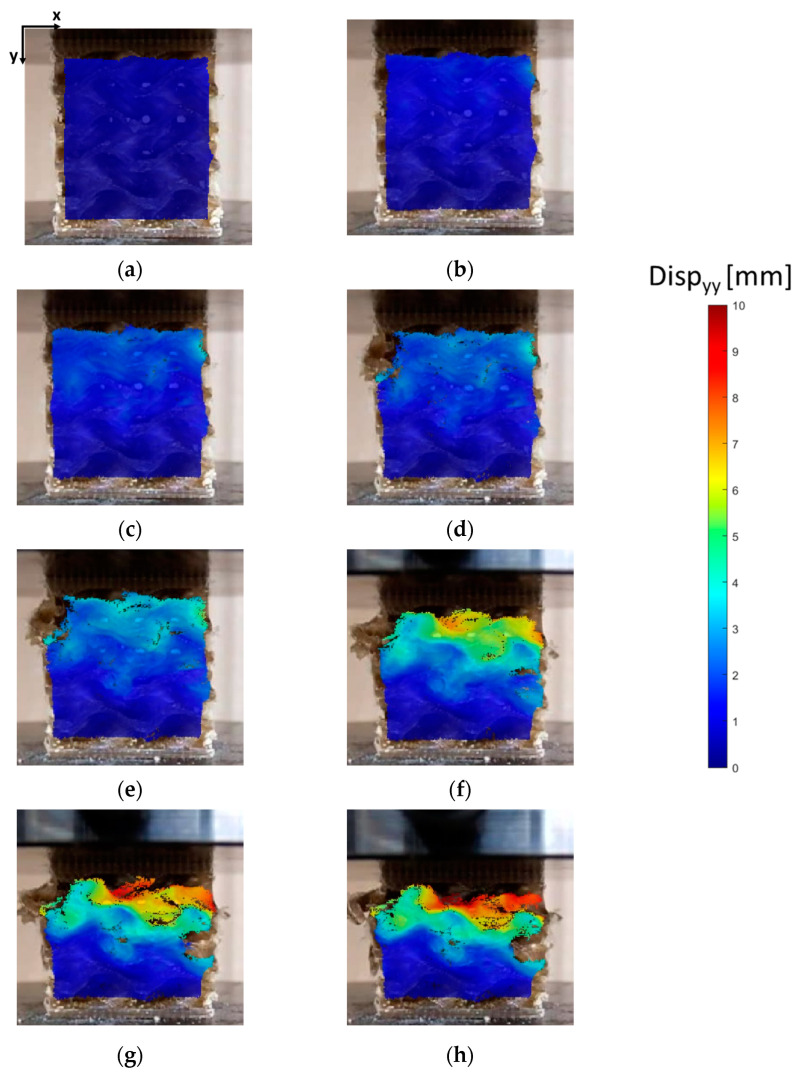
Vertical displacements calculated using the DIC technique for the specimen subjected to a compression test at 2 mm/min. The panels from (**a**–**c**) represent the distribution of displacement during the elastic phase. Image in panel (**d**) shows the displacement distribution at the load drop after the maximum load was reached, (**e**,**f**) show the displacements during the plateau region. Finally, images in panels (**g**,**h**) are related to the densification phase. Strain distributions confirm the collapsing mechanism described above, since the structure densifies along specific layers following the first macroscopic collapse of the gyroid strut, as shown in Figure 10.

**Figure 10:**
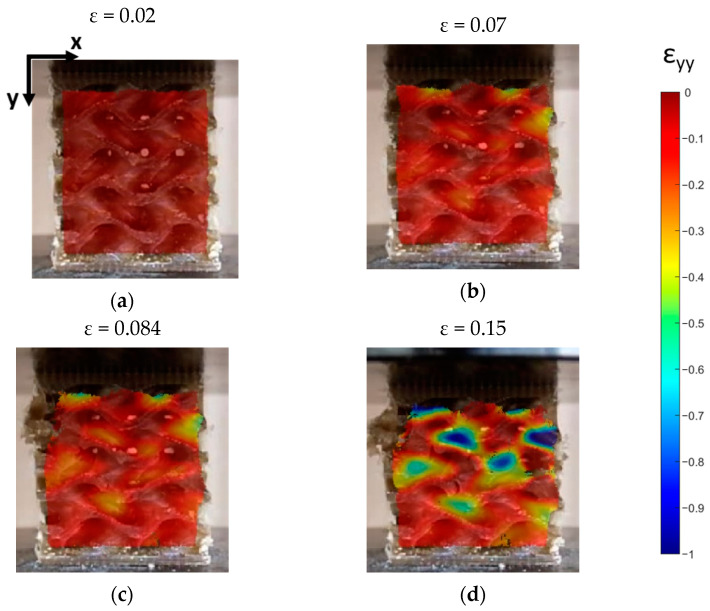
Vertical strain distribution calculated by DIC for the specimen subjected to compression test at 2 mm/min. The images refer to the following phases: (**a**) elastic phase; (**b**) strain at maximum load; (**c**) strain at the load drop after the maximum load; (**d**) plateau region.

Figure 8 shows that the curves present a linear elastic region followed by a plateau region. These plateau regions correspond to the collapse of the unit cells that characterize the lattice of the gyroid. The final rising slope of stress/strain curves is produced by the specimens’ densification, as depicted in DIC images (Figure 9g). DIC allows us to better understand the collapse mechanism of the gyroid structures. Figure 9 and Figure 10 show, respectively, the displacements and the strains in the direction of the loads application for the specimen tested at 2 mm/min.

Displacement values are mainly dependent on the curvature of the structure, and in the elastic region, they are almost constant for a given layer (Figure 9a–c). In the plastic region, after the first macroscopic collapse of the gyroid struts (Figure 9d), a plateau region with almost constant stress is observed (Figure 9e,f). In the last phase, the lattice experiences densification in specific layers, and the displacement values are no longer constant on a specific layer, as observed in the DIC images in Figure 9g,h.

In the Supplementary Materials, a graph with strain vs. time curves was reported for three different layers within the specimen (Figure S4). The graph highlights the collapsing mechanism described above: the three zones show the same strains up to the first macroscopic failure of the structure; on the contrary, values diverge in the plastic region, leading to a layer-by-layer collapse.

The mechanical properties of lattice specimens from compressive tests are reported in Table 3.

The failure modes, coming from consecutive layers collapsing, are in agreement with those reported by Spece et al. [51]. However, the mechanical properties cannot be compared between these two studies due to significant differences between their constitutive materials, porosity (55% in the current study vs. 74%), sample size (30 mm in the current study vs. 15 mm), and the presence of two end plates in the specimens built in this project. In detail, the yield stress assessed in the present study was systematically lower (11.03–12.21 MPa vs. 14.1–15.5 MPa), while the elastic modulus was significantly higher (301.5–309.6 vs. 210 ± 39.1 MPa).

Similar results can be found in [60], where the reported yield stress was 12.30 MPa, and the compression modulus was equal to 269.72 MPa for a specimen with a porosity equal to 42% and a sample size equal to 10 mm, even though these authors employed a different technology based on laser powder bed fusion.

### 3.3. Nanoindentation Tests

**Figure 11:**
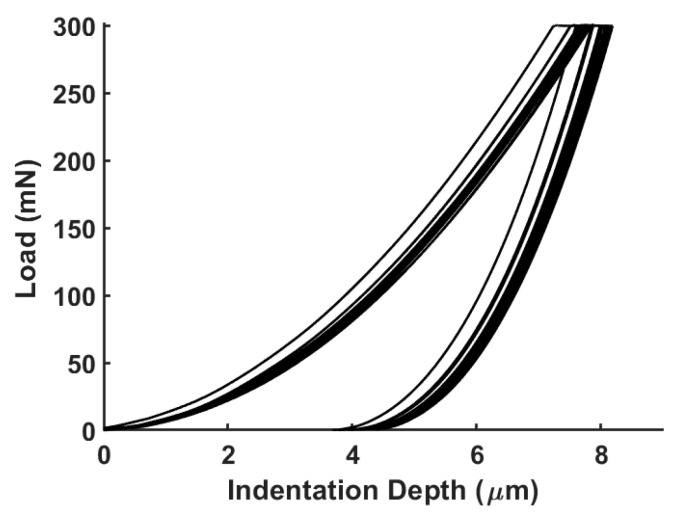
Load–displacement curves obtained from nanoindentation tests. Loading/unloading rates were set at 15 mN/s, imposing 20 s of holding time at the prescribed load value.

The experimental curves obtained from nanoindentation tests are depicted in Figure 11. Given the absence of pop-in features during the loading phase of the indentation curves, it can be assumed that no crack occurred during the tests [61]. As a matter of fact, the SEM analysis reported below confirms the absence of cracks at the apex of the indentation imprints, which is a typical feature of brittle materials [62].

The initial linear portion of the unloading phase of the nanoindentation curves corresponds to elastic deformations, where the material returns to its original shape upon unloading. The subsequent non-linear portion indicates plastic deformation, where permanent indentation occurs. In fact, the obtained nanoindentation curves demonstrate the elastoplastic nature of the tested material at the nanoscale. The presence of plasticity suggests that the material also exhibits some level of ductility at the nanoscale, resulting in permanent indentation. Furthermore, the holding period of 20 s was found to be sufficient in preventing the influence of viscous phenomena (absence of a ‘nose’ at the end of the holding phase [35]) during the unloading phase. 

The mean values of the nanoindentation modulus and nanoindentation hardness were determined to be 4.27 GPa (±0.085) and 0.030 GPa (±0.0086), respectively. The mean values of the nanoindentation modulus were found to be in accordance with values found in the literature for PEEK samples [63,64] and are also closer to the value of 5.1 GPa, obtained for tests on PEKK massive materials [43,57] Unfortunately, it was not possible to find more data concerning PEKK behavior at the nanoscale in the literature.

**Figure 12:**
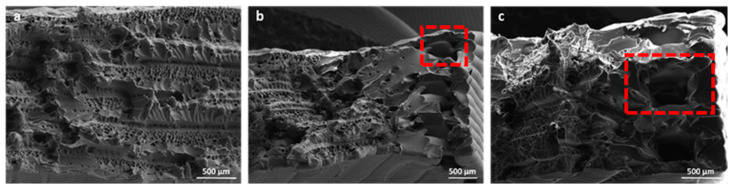
Explanatory SEM images of the fractured surfaces: (**a**,**b**) from specimen tested at 0.5 mm/min and (**c**) from specimen tested at 10 mm/min. Red boxes indicate the possible crack initiation site. Original images, from Figures S1–S3, are available in the Supplementary Materials.

**Figure 13:**
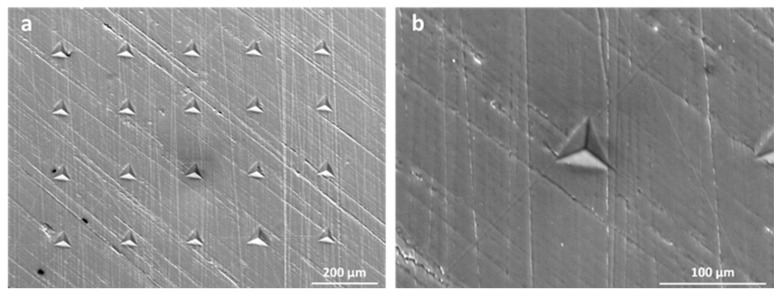
SEM images of nanoindentation imprints: (**a**) 4 × 5 indentation matrix; (**b**) representative indentation imprint.

Comparing the elastic modulus values obtained at the microscale through nanoindentation tests and at the macroscale through tensile tests, a significant difference was found (*p* < 10^−7^, Welch’s *t*-test) of about 1.71 GPa (+67%). The differences at these two scales can be attributed to the presence of voids, as observed in Figure 12. Furthermore, other studies have revealed that the mechanical properties obtained at the microscale, which are strongly influenced by the microstructural features of the material under analysis, may differ from the mechanical properties at higher scale levels [65,66]. The testing area for nanoindentation, as shown in Figure 13, was characterized by the absence of defects thanks to the polishing process required for nanoindentation tests. As a matter of fact, the mean value of the nanoindentation modulus was found to be in accordance with the elastic modulus found at the macroscale for molded PEEK, which was equal to 0.51 GPa [67]. On the contrary, the microstructure of the extruded PEKK shown in Figure 12 is characterized by the presence of defects that weaken the material at higher scales [67].

### 3.4. SEM Analysis

SEM analysis of the fractured surfaces of PEKK samples tested through uniaxial tensile tests is depicted in Figure 12. SEM analysis highlighted the presence of voids in the microstructure of the samples, probably generated during the extrusion process.

SEM analysis of the fractured samples highlighted a combination of smooth and rough regions on the fracture surface. Smooth areas indicate fast crack propagation, while rough areas suggest slower, more tortuous crack growth due to the presence of voids.

Figure 12a highlights the presence of a plastic flow that dominates the fracture process. The smooth area near the edge of the sample (red box in Figure 12b) and the squared hole (red box in Figure 12c), resulting from the deposition process, indicate the possible region where the crack originated followed by its fast propagation. The delamination process, characterized by the presence of little protrusions (Figure 12a,c), can be attributed to plastic deformations that develop during the necking phase of the tensile test. Furthermore, the reduced size of protrusions also indicates moderate delamination between adjacent layers, highlighting a strong interlayer bond able to withstand the applied stresses [68].

Figure 13 shows the area investigated by nanoindentation. Analysis of post-indentation features reveals the deformation mechanism of PEEK; the indentation imprints are characterized by a deformation mechanism known as pile-up that typically suggests ductile properties of the tested material at the nanoscale level [69].

## 4. Conclusions

In this study, a multiscale approach was implemented to experimentally evaluate the mechanical and geometrical properties of 3D-printed PEKK specimens. At the macroscale, both tensile and compression tests were performed on dog bone and gyroid specimens, respectively. Instead, at the nanoscale, nanoindentation tests were performed. Finally, micro-CT and confocal microscopy analyses were carried out, providing information on the surface and internal structure of specimens, while SEM analysis was performed to evaluate fracture mechanics.

Surface analyses performed by confocal microscopy illustrated the effect of the additive manufacturing process on the specimen, showing surface roughness along different directions, characterized by the presence of pronounced valleys between adjacent filaments.

Tensile tests showed that the mechanical properties of PEKK specimens produced through additive manufacturing are lower compared to those produced through conventional manufacturing techniques, in fact the respective a tensile strength has resulted to be 70.4 ± 4.5 MPa (in this study) versus 93 MPa [2]. The influence of loading conditions has been evaluated, and the results proved that the elastic modulus and ultimate tensile strength have low sensitivity to strain rate.

Nanoindentation allowed us to obtain useful information on the elastic and plastic properties of the constitutive material used to manufacture the analyzed specimens at the macroscale. Results obtained at different scales were found to be significantly different due to defects that characterize the microstructure of the analyzed specimens, thus highlighting the relevance of accurately replicating the microstructure, with its unavoidable defects, when studying the behavior of 3D-printed manufacts through numerical modeling technique. These experimental data, along with SEM and micro-CT results, provide useful information that will be used in future works to define and implement a numerical model (finite elements) capable of replicating the actual behavior of 3D-printed PEKK both globally and locally.

## Figures and Tables

**Table 1:** Detailed analysis of surface properties.

Parameter	Upper Surface	Lateral Surface
S_q_ ^1^ [μm]	18.76	26.83
S_sk_ ^2^	−0.31	−0.86
S_ku_ ^3^	3.04	2.23
S_p_ ^4^ [μm]	49.23	66.23
S_v_ ^5^ [μm]	70.34	60.12
S_z_ ^6^ [μm]	119.57	126.35
S_a_ ^7^ [μm]	15.12	22.96

^1^ root mean square height of the surface; ^2^ skewness of the height distribution; ^3^ kurtosis of the height distribution; ^4^ maximum height of peaks; ^5^ maximum height of valleys; ^6^ maximum height of the surface; ^7^ mean height of the surface.

**Table 2:** Mechanical properties of the specimens subjected to tensile tests.

	Loading Rate					
Properties	0.5 mm/min	1 mm/min	2 mm/min	5 mm/min	10 mm/min	100 mm/min
Elastic Modulus E [MPa]	2602	2312	2603	2634	2456	2728
Tensile Strength σ_m_ [MPa]	67.35	64.40	70.50	70.66	72.06	77.57
Elongation at break ε_u_ [%]	11.14	8.39	8.28	8.04	8.53	10.20

**Table 3:** Mechanical properties of lattice specimens from compressive tests.

Properties	0.5 mm/min	2 mm/min	5 mm/min	10 mm/min
DIC Elastic Modulus E_DIC_ [MPa]	309.6	305.7	301.5	308.4
Compressive Strength σ_c_ [MPa]	11.36	11.59	11.03	12.21
Deformed Shape	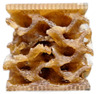	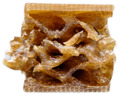	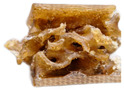	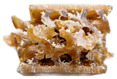

## Data Availability

Data will be shared upon request to the corresponding authors.

## Supplementary Materials

The following supporting information can be downloaded at: https://www.mdpi.com/article/10.3390/bioengineering11030244/s1, Figure S1. Explanatory SEM image of fractured surface from specimen tested at 0.5 mm/min. Figure S2. Explanatory SEM image of fractured surface from specimen tested at 0.5 mm/min. Figure S3. Explanatory SEM image of fractured surface from specimen tested at 10 mm/min. Figure S4. (a) Global Stress-Time curve of the compressive specimen; Local Strain-time curves of the compressive specimen referred to: (b) zone 1, zone 2 and zone 3.
