# Supplementary material for: Multiscale Mechanical Characterization of Polyether-2-ketone (PEKK) for Biomedical Application
Source: Bioengineering (Basel). 2024 Feb 29;11(3):244. doi: 10.3390/bioengineering11030244 (PMC10968480; doi:10.3390/bioengineering11030244)
Supplement: Supplementary file 1 [file bioengineering-11-00244-s001.zip › bioengineering-2811583-supplementary.pdf]

Supplementary Material

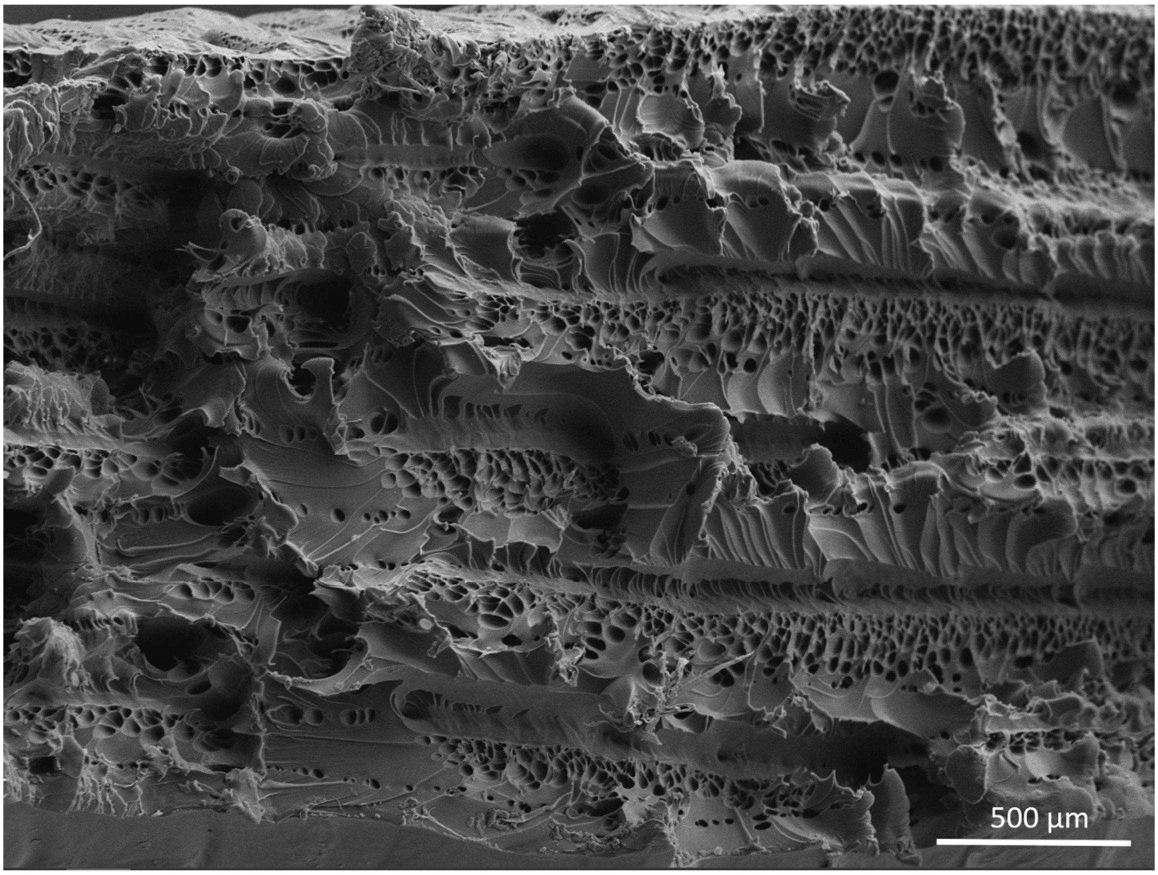

Figure S1. Explanatory SEM image of fractured surface from specimen tested at 0.5 mm/min.

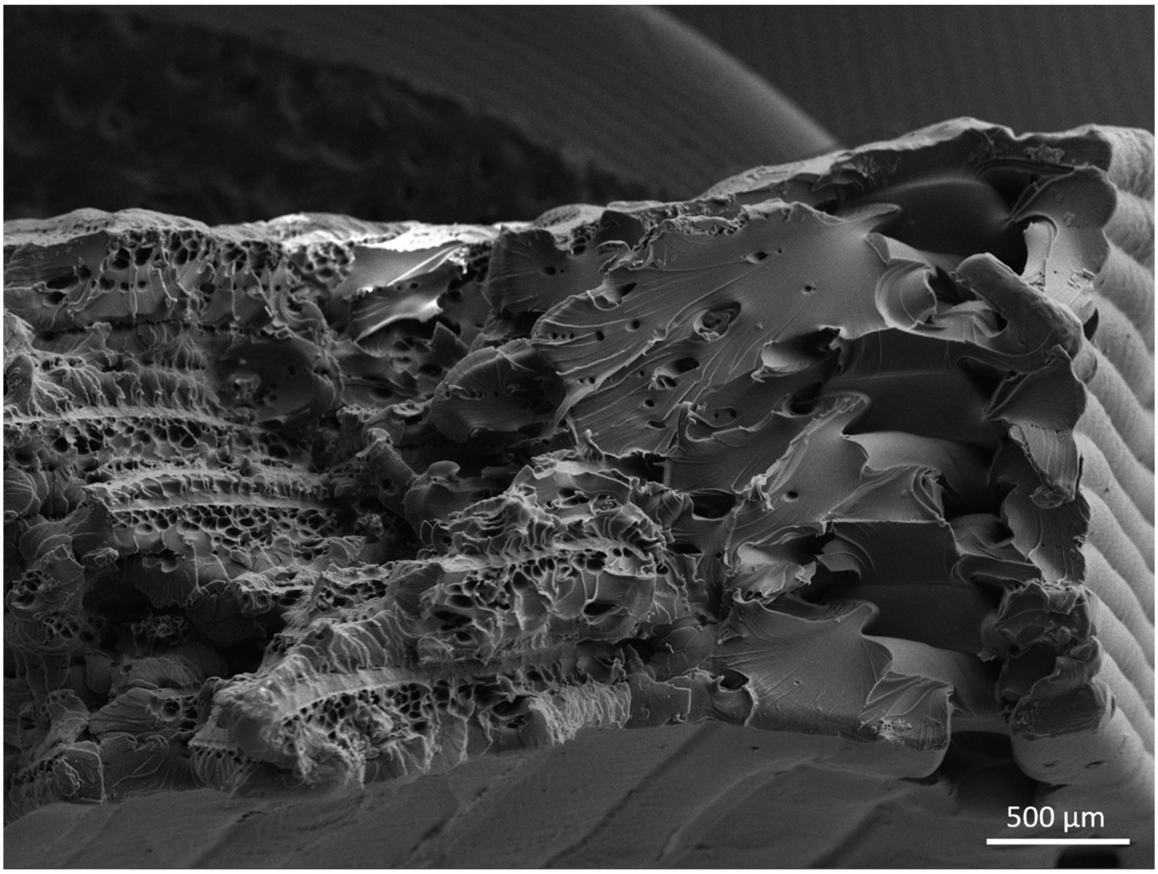

Figure S2. Explanatory SEM image of fractured surface from specimen tested at 0.5 mm/min.

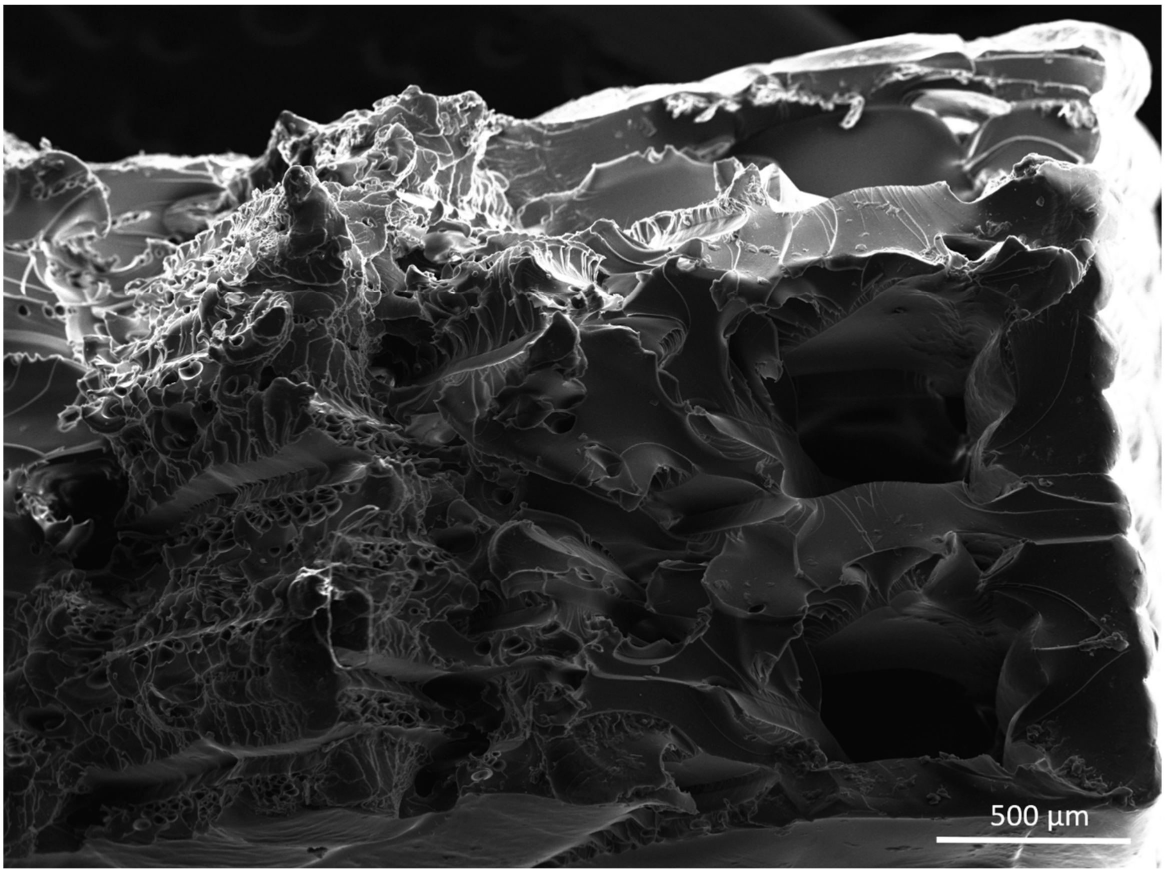

Figure S3. Explanatory SEM image of fractured surface from specimen tested at 10 mm/min.

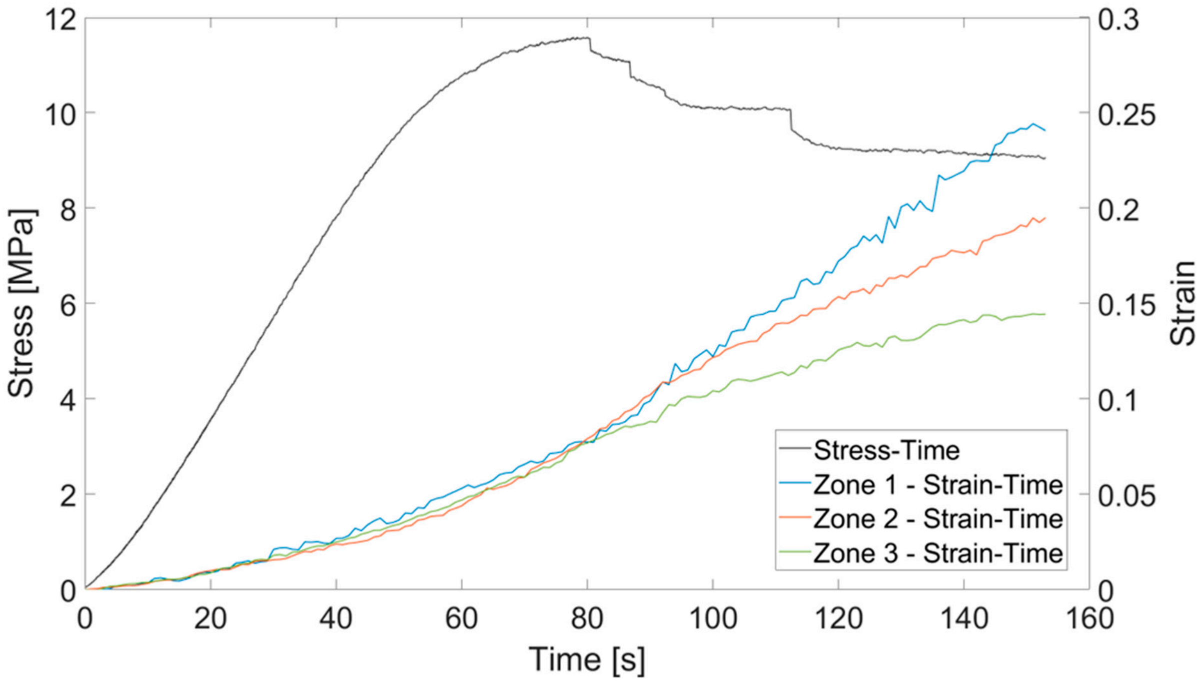

(a)

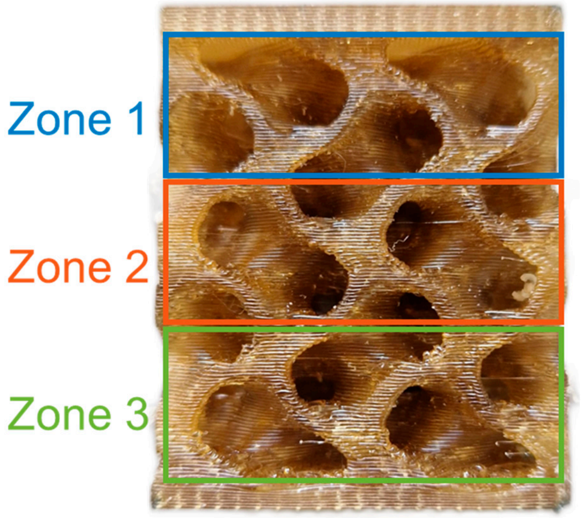

(b)

Figure S4. (a) Global Stress-Time curve of the compressive specimen; Local Strain-time curves of the compressive specimen referred to: (b) zone 1, zone 2 and zone 3
